# PEPTERGENT: A Peptide-Based Reagent for Detergent-Free Extraction of Membrane Proteins and Purification of Membrane Proteomes

**DOI:** 10.21769/BioProtoc.5700

**Published:** 2026-05-20

**Authors:** Frank Antony, Ashim Bhattacharya, Franck Duong van Hoa

**Affiliations:** Department of Biochemistry and Molecular Biology, Faculty of Medicine, Life Sciences Institute, University of British Columbia, Vancouver, BC, Canada

**Keywords:** Detergent, Peptidisc, Nanodisc, SMA, DeFrND, 18A peptide, Membrane mimetic, Protein structure, Proteomics

## Abstract

Peptergent is a novel class of amphipathic peptides that enables detergent-free extraction of membrane proteins (MPs) from lipid bilayers. This reagent self-assembles around hydrophobic transmembrane regions, forming stable, water-soluble complexes that can be isolated directly from biological membranes. Peptergent therefore bypasses the limitations imposed by traditional detergents, which often destabilize protein assemblies. Since detergents are completely avoided, MPs are directly amenable to structural and mass spectrometry (MS) analysis, thereby addressing their persistent underrepresentation in proteomic datasets and improving their accessibility in drug-screening strategies. We present here a streamlined protocol for MPs extraction with the Peptergent PDET-1, followed by exchange into His-tagged Peptidiscs for Ni-NTA-based affinity purification. The method encompasses membrane isolation, peptide preparation, protein extraction, clarification, and MPs exchange from Peptergents to Peptidiscs. This workflow yields an enriched membrane proteome compatible with downstream LC-MS/MS analysis for improved identification of multi-pass MPs.

Key features

• Direct extraction and solubilization of membrane proteins.

• 100% detergent-free workflow.

• Exchange of Peptergent to Peptidiscs for affinity purification of membrane proteins.

• Applicable to cultured cells and tissue-derived membrane fractions.

## Graphical overview


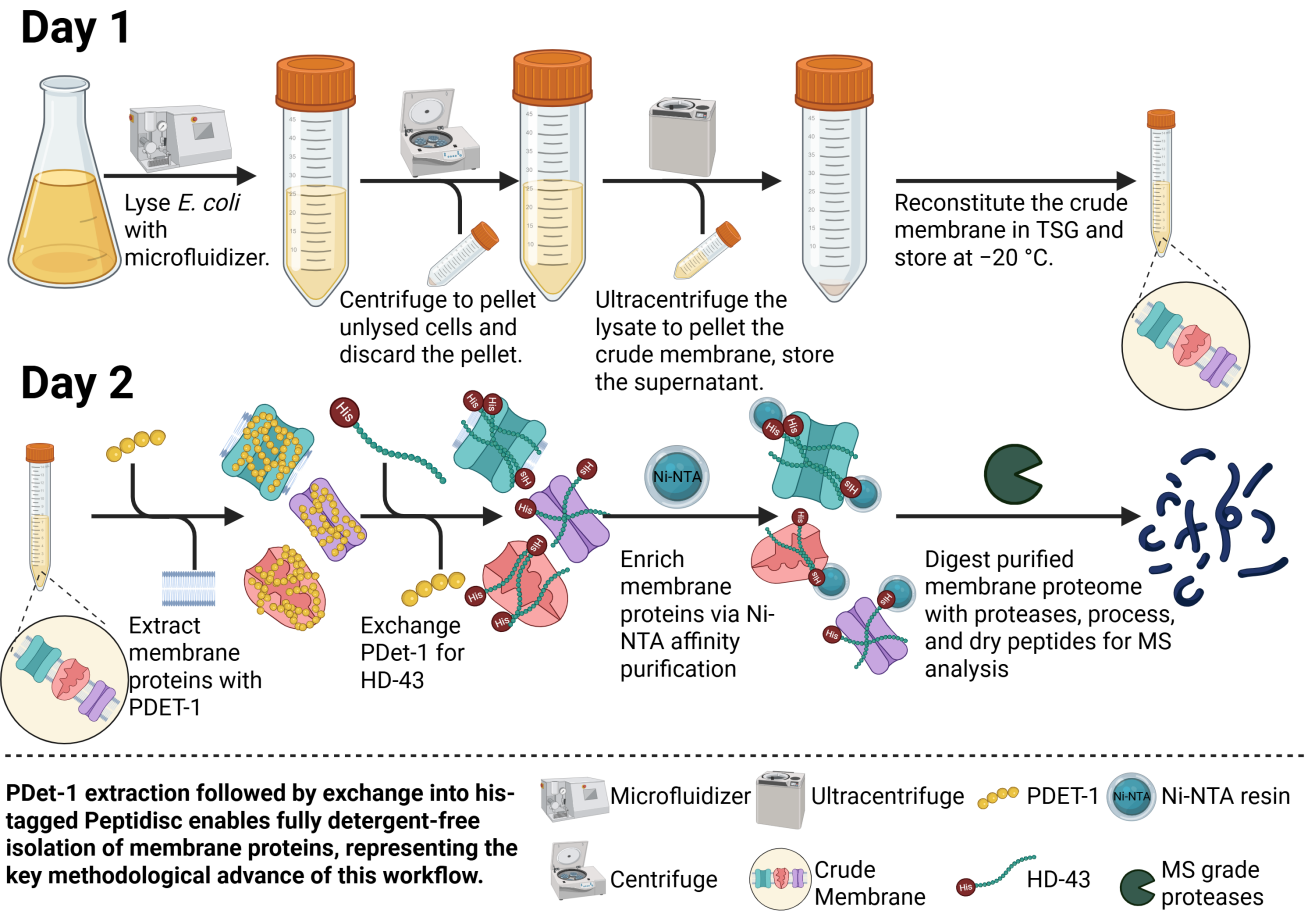


**Peptergent-based workflow for isolating membrane proteins (MPs) directly from membranes.** Proteins are extracted with Peptergent (PDET-1) and transferred into His-tagged Peptidisc (HD-43). The water-soluble MPs are purified by Ni-NTA affinity purification before preparation for bottom-up mass spectrometry. The protocol yields dried peptide samples ready for LC–MS analysis.

## Background

Membrane proteins (MPs) constitute nearly ~30% of the proteome and represent the majority of current therapeutic targets [1]. Yet, their identification and biochemical and structural characterization remain fundamentally constrained by the need for extraction from lipid bilayers while preserving structure, oligomeric integrity, and ligand responsiveness [2–4]. Conventional workflows rely on detergent membrane solubilization followed by protein purification in mixed micelles, a process that frequently destabilizes MPs, disrupts native lipid interactions, and biases downstream structural and functional analyses [3,5,6]. These surfactants are also mostly incompatible with downstream mass spectrometry (MS), thereby altering the precision of quantitative membrane proteome analysis [7].

Detergent-free systems, including nanodiscs, Peptidiscs, and styrene–maleic acid (SMA), partially address these limitations [8]. However, these membrane mimetic approaches still require an initial detergent extraction, while the polymer-based SMA introduces abundant ionic charges that necessitate organic-phase extraction [8,9]. Consequently, a gap remains between native membrane isolation and MS-compatible analysis, such as enabling direct solubilization of the membrane proteome without prior detergent exposure or polymer-induced modification.

Previous and more recent studies have demonstrated that short 18A-based amphiphilic peptides can solubilize membrane proteins from lipid bilayers in a manner analogous to conventional detergents [10,11]. Because these peptides combine detergent-like extraction properties with a peptide-based scaffold, they have been termed peptide detergents, or “Peptergents” [12]. Early studies showed that Peptergents efficiently solubilize membrane proteins while improving the stability and longevity of membrane enzymes compared with traditional surfactants [10,12–15]. Structural studies and molecular modeling later suggested that these peptides self-assemble into ordered supramolecular architectures, potentially adopting a β-sheet-like organization that solubilizes lipids and encircles hydrophobic protein surfaces to essentially mimic the classical detergent micelles action [16,17].

Building on these earlier discoveries, we describe the use of the Peptergent PDET-1 for detergent-free extraction of MPs from biological membranes, enabling downstream MS analysis. Membranes are incubated with PDET-1, forming peptide-stabilized MP assemblies. Because PDET-1 lacks an affinity handle, solubilized MPs are subsequently exchanged into His-tagged Peptidiscs, enabling Ni-NTA affinity purification and depleting soluble contaminants that co-extract during membrane solubilization [18–22]. This workflow shows membrane isolation, peptide preparation, PDET-1 extraction, exchange into His-tagged Peptidiscs, Ni-NTA-based enrichment, and bottom-up MS sample preparation. This detergent-free process can be applied to crude membrane fractions from cultured cells and tissue fractions.

## Materials and reagents

**Biological materials**

1. Glycerol stock of Bl21 (DE3) competent cells *E. coli* (originally purchased from Millipore Sigma, catalog number: 69450-M); check Addgene (https://www.addgene.org/protocols/create-glycerol-stock/) for preparation instructions and store at -80 °C

*Note: No antibiotic selection is used in this protocol, as cells are grown without a plasmid for crude membrane isolation.*

**Reagents**

1. PDET-1 (Peptergent); peptide fragment derived from the Peptidisc peptide (Peptidisc Biotech); store at -20 °C

2. HD-43 (His-Tagged Peptidisc); His-branch on the Peptidisc peptide; Peptidisc Biotech; store at -20 °C

3. Ni-NTA agarose (Qiagen, catalog number: 30210); store at 4 °C

4. cOmplete protease inhibitor cocktail (Sigma-Aldrich, catalog number: 11697498001); store at 4 °C

5. MS-grade proteases (Thermo Fisher Scientific, catalog number: 90057); store at -20 °C

6. C18-HD disk 47 mm; Empore (C18 solid phase extraction disk) (UNSPSC, catalog number: 41120000); store at room temperature

7. Polygoprep 300-20 C18 (C18 resin) (Macherey-Nagel, catalog number: 711025.100); store at room temperature

8. Tris (BioShop, catalog number: TRS003.5); store at room temperature

9. Sodium chloride (NaCl) (Fisher Scientific, catalog number: BP358-212); store at room temperature

10. Ethylenediaminetetraacetic acid (EDTA) (BioShop, catalog number: EDT002.500); store at room temperature

11. Phenylmethylsulfonyl fluoride (PMSF) (Sigma-Aldrich, catalog number: P-7626); store at room temperature

12. Dithiothreitol (DTT) (BioShop, catalog number: DTT002.10); store at -20 °C

13. Iodoacetamide (IAA) (BioShop, catalog number: IOD500.5); store at 4 °C

14. Urea (BioShop, catalog number: URE002.5); store at room temperature

15. Methanol (Sigma-Aldrich, catalog number: 179337-4L); store at room temperature

16. Acetonitrile (certified ACS) (Fisher Scientific, catalog number: A211); store at room temperature

17. Imidazole (BioShop, catalog number: IMD510.500); store at room temperature

18. Formic acid (Sigma-Aldrich, catalog number: 695076-100ML); store at room temperature

19. Tryptone (Bioshop, catalog number: TRP402.5); store at room temperature

20. Yeast extract (Bioshop, catalog number: YEX555.500); store at room temperature

21. Aluminum foil (Canada Tire, catalog number: 053-0371-0); store at room temperature

22. Glycerol (Fisher Scientific, catalog number: BP229-4); store at room temperature

23. Ammonium bicarbonate (Fisher Scientific, catalog number: A643500); store at room temperature

24. Sodium hydroxide (NaOH) (Fisher Scientific, catalog number: S318-3); store at room temperature

25. Hydrochloric acid (HCl) solution (Fisher Scientific, catalog number: A144-212); store at room temperature

26. Bleach; Lavo Pro 6 (ULINE Canada, catalog number: S-24408); store at room temperature

27. 100% reagent alcohol (Fisher Scientific, catalog number A962^F^-1Gal); store at room temperature

*Note: This reagent is regulated and requires purchase and tracking through the university.*

28. Medical-grade compressed air in K-sized cylinders [please contact Linde directly to inquire how to purchase this item (1-800-225-8247)]

29. Water suitable for HPLC (Sigma-Aldrich, catalog number: 270733-1L); store at room temperature

30. Protein assay dye reagent concentrate (Bio-Rad, catalog number: 5000006); store at 4 °C

31. Precision Plus protein unstained protein standards (Bio-Rad, catalog number: 1610363); store at -20 °C

**Solutions**

1. 6 M NaOH solution (see Recipes)

2. 6 M HCl solution (see Recipes)

3. 20% ethanol solution (see Recipes)

4. 1 M Tris pH 7.8 solution (see Recipes)

5. 1 M NaCl solution (see Recipes)

6. 50% glycerol solution (see Recipes)

7. TS buffer (see Recipes)

8. 2× TS buffer (see Recipes)

9. TSG buffer (see Recipes)

10. LB media (see Recipes)

11. 50 mM ammonium bicarbonate solution (see Recipes)

12. 2 M imidazole solution (see Recipes)

13. Ni-NTA elution buffer (see Recipes)

14. 1 M PMSF solution (see Recipes)

15. 0.5 M EDTA pH 8.0 solution (see Recipes)

16. 100× protease inhibitor tablet solution (see Recipes)

17. 0.5 mg/mL mass spec protease solution (see Recipes)

18. 1 M DTT solution (see Recipes)

19. 500 mM IAA solution (see Recipes)

20. 10 mg/mL PDET-1 solution (see Recipes)

21. 5 mg/mL HD-43 solution (see Recipes)

22. Solubilization buffer (see Recipes)

23. C18 resin slurry (see Recipes)

24. 10% formic acid solution (see Recipes)

25. 0.1% formic acid solution (see Recipes)

26. 40% acetonitrile, 0.1% formic acid solution (see Recipes)

27. 1× Bradford assay solution (see Recipes)

**Recipes**

A useful formula for determining the amount of reagent to weigh when preparing a solution:

Grams to weigh = desired molarity (mol/L) * molecular weight (g/mol) * desired volume (L)

Use an online molarity calculator to determine adjusted values if using different quantities while preparing reagents (https://www.calculator.net/molarity-calculator.html).

**1. 6 M NaOH solution (250 mL)**

**Table:** 

Reagent	Final concentration	Quantity or volume
NaOH (39.997 g/mol)	6 M	60 g
Distilled water	NA	To 250 mL

Store at room temperature.

**2. 6 M HCl solution (250 mL)**

**Table:** 

Reagent	Final concentration	Quantity or volume
Commercially purchased 12.1 M HCl	6 M	124 mL
Distilled water	NA	126 mL

Store at room temperature.

**3. 20% ethanol solution (250 mL)**

**Table:** 

Reagent	Final concentration	Quantity or volume
Commercially purchased 100% ethanol solution	20%	50 mL
Distilled water	NA	200 mL

Store at room temperature.

**4. 1 M Tris pH 7.8 solution (500 mL)**

**Table:** 

Reagent	Final concentration	Quantity or volume
Tris (121.14 g/mol)	1 M	60.57 g
6 M HCl solution		to pH 7.8
Distilled water	NA	To 500 mL

Store at room temperature.

**5. 1 M NaCl solution (1 L)**

**Table:** 

Reagent	Final concentration	Quantity or volume
NaCl (58.44 g/mol)	1 M	58.44 g
Distilled water	NA	To 1 L

Store at room temperature.

**6. 50% glycerol solution (200 mL)**

**Table:** 

Reagent	Final concentration	Quantity or volume
Commercially purchased 100% glycerol solution	50%	100 mL
Distilled water	NA	100 mL

Avoid using a serological pipette or graduated cylinder as the glycerol will stick to the walls. It is more accurate to slowly pour the solution into the media bottle. Store at room temperature.

**7. TS buffer (1 L)**

**Table:** 

Reagent	Final concentration	Quantity or volume
1 M Tris pH 7.8 solution	50 mM	50 mL
1 M NaCl solution	100 mM	100 mL
Distilled water	NA	850 mL

Store at room temperature or 4 °C.

**8. 2× TS buffer (1 L)**

**Table:** 

Reagent	Final concentration	Quantity or volume
1 M Tris pH 7.8 solution	100 mM	100 mL
1 M NaCl solution	200 mM	200 mL
Distilled water	NA	700 mL

Store at room temperature or 4 °C.

**9. TSG buffer (1 L)**

**Table:** 

Reagent	Final concentration	Quantity or volume
1 M Tris pH 7.8 solution	50 mM	50 mL
1 M NaCl solution	100 mM	100 mL
50% glycerol solution	10%	200 mL
Distilled water	NA	650 mL

Store at room temperature or 4 °C.

**10. LB media (1 L)**

**Table:** 

Reagent	Final concentration	Quantity or volume
Tryptone (no specified molecular weight)	NA	10 g
Yeast extract (no specified molecular weight)	NA	5 g
NaCl (58.44 g/mol)	171 mM	10 g
Distilled water	NA	To 1 L

Autoclave at 120 °C for 30 min with a small volume of water in the autoclave tray. Ensure the lid is loosely fit and the autoclave tape is wrapped around the top of the bottle before autoclaving. After autoclaving, store at room temperature. If anything is seen growing inside, add bleach at a volume of 10% of the combined LB media and bleach volume and mix. Let it sit for 30 min at room temperature. Then pour down the drain and chase the drain with tap water for 15 min.

**11. 50 mM ammonium bicarbonate solution (50 mL)**

**Table:** 

Reagent	Final concentration	Quantity or volume
Ammonium bicarbonate (79.06 g/mol)	50 mM	197.65 mg
Distilled water	NA	Up to 50 mL

Store at 4 °C.

**12. 2 M imidazole solution (50 mL)**

**Table:** 

Reagent	Final concentration	Quantity or volume
Imidazole (68.08 g/mol)	2 M	6.8 g
Distilled water	NA	Up to 50 mL

Store at 4 °C.

**13. Ni-NTA elution buffer (10 mL)**

**Table:** 

Reagent	Final concentration	Quantity or volume
2× TS	50 mM Tris pH 7.8, 100 mM NaCl	5 mL
2 M imidazole solution	600 mM imidazole	3 mL
Distilled water	NA	2 mL

Store at 4 °C.

**14. 1 M PMSF solution (10 mL)**

**Table:** 

Reagent	Final concentration	Quantity or volume
PMSF (174.19 g/mol)	1 M	1.74 g
Isopropanol	NA	Up to 10 mL

Store at -20 °C. A precipitate may form during storage; use only the clear supernatant and avoid the insoluble fraction. Prepare fresh stock once the soluble portion is depleted.

**15. 0.5 M EDTA pH 8.0 solution (50 mL)**

**Table:** 

Reagent	Final concentration	Quantity or volume
EDTA (372.24 g/mol)	0.5 M	9.3 g
6 M NaOH solution		to pH 8.0
Distilled water	NA	Up to 50 mL

Dissolve 1.3 g of EDTA in 30 mL of distilled water. Adjust the pH to 8.0 using 6 M NaOH. Add an additional 1 g of EDTA, mix until dissolved, and re-adjust the pH to 8.0. Continue adding 1 g of EDTA at a time, adjusting the pH back to 8.0 after each addition, until a total of 9.3 g of EDTA has been added. Bring the final volume to 50 mL with distilled water. Store at 4 °C.

**16. 100× protease inhibitor tablet solution (100 μL)**

**Table:** 

Reagent	Final concentration	Quantity or volume
cOmplete protease inhibitor cocktail	100×	1 tablet
Distilled water	NA	500 μL

Store at -20 °C unless using the day of.

**17. 0.5 mg/mL mass spec protease solution (40 μL)**

**Table:** 

Reagent	Final concentration	Quantity or volume
Commercially purchased MS-grade proteases	0.5 μg/μL	20 μg
1 M Tris pH 7.8 solution	NA	40 μL

Store at -20 °C. If the protease mix is used within 1 h, it may be kept on ice instead of freezing.

**18. 1 M DTT solution (100 μL)**

**Table:** 

Reagent	Final concentration	Quantity or volume
DTT (154.25 g/mol)	1 M	15.42 mg
HPLC-grade water	NA	100 μL

Prepare the solution fresh on the day of use and keep on ice until needed.

**19. 500 mM IAA solution (100 μL)**

**Table:** 

Reagent	Final concentration	Quantity or volume
IAA (184.96 g/mol)	500 mM	9.2 mg
HPLC-grade water	NA	100 μL

Prepare the solution fresh immediately before use, keep on ice, and protect it from light by keeping the tube covered or wrapped in foil when not in use.

**20. 10 mg/mL PDET-1 solution (2 mL)**

**Table:** 

Reagent	Final concentration	Quantity or volume
Commercially purchased PDET-1	10 mg/mL	20 mg
1 M Tris pH 7.8 solution	pH 7.5	X mL
Distilled water	NA	To 2 mL

Store at 4 °C. Check pH with pH indicator strips 5.0–10.0.

**21. 5 mg/mL HD-43 solution (4 mL)**

**Table:** 

Reagent	Final concentration	Quantity or volume
Commercially purchased HD-43	5 mg/mL	20 mg
1 M Tris pH 7.8 solution	pH 7.5	X mL
Distilled water	NA	To 4 mL

Store at 4 °C. Check pH with pH indicator strips 5.0–10.0.

**22. Solubilization buffer (4 mL)**

**Table:** 

Reagent	Final concentration	Quantity or volume
10 mg/mL PDET-1 solution	5 mg/mL	2 mL
1 M Tris pH 7.8 solution	50 mM	200 μL
1 M NaCl solution	100 mM	400 μL
0.5 M EDTA pH 8.0 solution	1 mM	8 μL
100× protease inhibitors solution	1×	40 μL
Distilled water	NA	To 4 mL

Make fresh and keep on ice for use on the same day.

**23. C18 resin slurry (600 μL)**

**Table:** 

Reagent	Final concentration	Quantity or volume
Commercially purchased C18 resin	NA	200 μL of resin
Methanol	NA	400 μL

Make just before use.

**24. 10% formic acid solution (10 mL)**

**Table:** 

Reagent	Final concentration	Quantity or volume
Commercially purchased 100% formic acid solution	10%	1 mL
HPLC-grade water	NA	9 mL

Store at 4 °C.

**25. 0.1% formic acid solution (10 mL)**

**Table:** 

Reagent	Final concentration	Quantity or volume
10% formic acid solution	0.1%	100 μL
HPLC-grade water	NA	9.9 mL

Store at 4 °C.

**26. 40% acetonitrile, 0.1% formic acid solution (10 mL)**

**Table:** 

Reagent	Final concentration	Quantity or volume
Commercially purchased 100% acetonitrile solution	40%	4 mL
10% formic acid solution	0.1%	100 μL
HPLC-grade water	NA	5.9 mL

Store at 4 °C.

**27. 1× Bradford assay solution (50 mL)**

**Table:** 

Reagent	Final concentration	Quantity or volume
Commercially purchased protein assay dye reagent concentrate	1×	10 mL
Distilled water	40 mL	40 mL

Store at 4 °C.

**Laboratory supplies**

1. 10 mL polystyrene serological pipets (Fisher Scientific, catalog number: 1367518)

2. 1.5 mL microcentrifuge tubes (Fisher Scientific, catalog number: 05408129)

3. Nitrile gloves (FroggaBio, catalog number: FG100M-10)

4. pH indicator strips 2.5–4.5 (Millipore-Sigma, catalog number: 1095410001)

5. pH indicator strips 5.0–10.0 (Millipore-Sigma, catalog number: 1095330001)

6. Ice (provided by the university)

7. Falcon 50 mL conical centrifuge tube (Fisher Scientific, catalog number: 1443222)

8. Falcon 15 mL conical centrifuge tube (Fisher Scientific, catalog number: 1495953B)

9. Needle (Becton Dickinson, catalog number: 305196)

10. Gel-loading tips (Fisher Scientific, catalog number: 02707181)

## Equipment

1. Burner (Fisher Scientific, catalog number: 01257557)

2. Laboratory lighter (Fisher Scientific, catalog number: NC169758815)

3. Pipette controller (Fisher Scientific, catalog number: 10-320-173)

4. 7 and 15 mL glass Douncer (Fisher Scientific, catalog numbers: K885300007 and K8853000015)

5. Eppendorf tabletop centrifuge 5415D (Sigma-Aldrich, catalog number: Z604062)

6. Tabletop Ultracentrifuge 130,000 RPM (Beckman Coulter, model: Optima Max)

7. TLA-55 fixed-angle rotor (Beckman Coulter, catalog number: 366725)

8. TLA-55 1.5 mL polypropylene tube with snap-on cap (Beckman Coulter, catalog number: 343169)

9. C24 incubator shaker (New Brunswick Scientific, model: C24)

10. C25 incubator shaker (New Brunswick Scientific, model: C25)

11. M110L microfluidizer processor (Microfluidics Corporation, model: M110L)

12. Labquake Rotisserie (ThermoFisher Scientific)

13. Corning 125 mL and 1 L Erlenmeyer flasks (Fisher Scientific, catalog number: 100414A and D)

14. Bulk p1000, p200, and p10 pipette tips (Sarstedt, catalog numbers: 70.1187.102, 70.3030.020, 70.3010.205)

15. Scoopula (Fisher Scientific, catalog number: 01257567)

16. Magnetic stir bars (Fisher Scientific, catalog number: 1451357SIX)

17. 100 mL, 250 mL, 500 mL, and 1 L media storage bottles (Fisher Scientific, catalog numbers: FB800100, FB800250, FB800500, FB8001000)

18. Avanti J-E Centrifuge (Beckman Coulter, model: Avanti J-E)

19. JA-25.50 fixed-angle rotor-aluminum (Beckman Coulter, catalog number: 363055)

20. JA 25.50 50 mL polypropylene bottle with cap assembly (Beckman Coulter, catalog number: 361694)

21. JLA10.500 fixed-angle aluminum rotor (Beckman Coulter, catalog number: 369681)

22. JLA10.500 500 mL polycarbonate bottles (Beckman Coulter, catalog number: 369681)

23. Genesys 10 UV-Vis spectrophotometer (Fisher Scientific, catalog number: 14385400)

24. Nutator (Clay Adams)

25. Analog vortex mixer (Fisher Scientific, catalog number: 02215414)

26. Optima LE-80k ultracentrifuge (Beckman Coulter, model: Optima LE-80k)

27. TI-45 fixed-angle titanium rotor (Beckman Coulter, catalog number: 339160)

28. TI-45 round-top ultra-clear tube (Beckman Coulter, catalog number: 345778)

29. Analytical scale (Denver Instruments Company, model: TL-64)

30. Toploading scale (Denver Instruments Company, model: APX-2001)

31. Hot plate stirrer (Corning, model: PC-351)

32. pH meter AB15 (Fisherbrand accumet, model: AB15)

33. Standard-duty vacuum pump (Fisher Scientific, model: 2545B-01)

34. Thermo Savant Speed-Vac Concentrator SC110 (Marshall Scientific, model: SC110)

35. Savant Speed-Vac Refrigerated Vapor Trap (Fisher Scientific, catalog number: 13875349)

36. Semi-micro cuvette (Millipore-Sigma, catalog number: BR759015-100EA)

37. 4 °C fridge and -20 °C freezer (Insignia, catalog number: NS-UZ17XWH7)

38. Ultra-low freezer (-80 °C) (Thermo Fisher Scientific, catalog number: TSX70086CA)

## Procedure

**A. *E. coli* culture growth and cell harvesting**

1. Activate the CO_2_-connected Bunsen burner by turning the valve to release gas, and ignite the burner using a lighter.

2. Near a lit Bunsen burner (to reduce the risk of contamination from airborne particles), add 50 mL of LB medium to a sterile 125 mL Erlenmeyer flask.

3. Remove the BL21 *E. coli* glycerol stock from the -80 °C freezer. Using a sterile P200 pipette tip, scrape a small frozen portion of the glycerol stock.

4. Drop the frozen scrape of the glycerol stock into the LB medium in the 125 mL Erlenmeyer flask while working near the Bunsen burner flame.

5. Turn off the Bunsen burner and incubate the Erlenmeyer flask containing the bacterial scrape in the C24 incubator shaker at 37 °C and 200 rpm for 12–16 h to generate the bacterial starter culture.

*Note: The bacterial starter culture should appear turbid/cloudy after overnight incubation, with an OD_600_ > 1.0.*

**Caution:** The LB medium does not contain antibiotics, so any contaminants introduced during handling will also grow in the culture. Perform all steps near an active Bunsen burner to maintain sterility.

6. The next day, ignite the Bunsen burner using a striker. Working near the flame, transfer LB medium into a 1 L Erlenmeyer flask and use the flask’s graduations to measure 1 L, as the medium cannot be transferred to a graduated cylinder due to the absence of antibiotics.

7. Remove the bacterial starter culture and, working near the flame, transfer 10 mL into the 1 L Erlenmeyer flask containing 1 L of fresh LB medium. Cover the mouth of the flask with aluminum foil to prevent contaminants from entering the culture during incubation.

8. Transfer the flask to the C25 incubator shaker and grow the culture at 37 °C with shaking at 200 rpm for 8–16 h.

9. After incubation, remove the bacterial culture from the C25 shaker. Transfer 500 mL of the culture into each of two JLA 10.500 polypropylene centrifuge tubes. Weigh the tubes to ensure they are balanced within 5 g of each other.

10. Place the JLA-10.500 rotor into the Avanti J-E centrifuge.

11. Place the two centrifuge tubes into JLA-10.500 biosafety liquid canisters and position them opposite each other in the JLA-10.500 rotor. Fill the remaining rotor positions with empty biosafety liquid canisters to ensure the rotor is fully balanced.

**Critical:** All slots in the JLA-10.500 rotor must be filled before centrifugation. Any unused positions must be occupied with empty biosafety liquid canisters to ensure proper rotor balance during the spin.

12. Centrifuge at 6,000× *g* for 15 min.

13. After centrifugation, discard the supernatant (spent LB media) down the drain, leaving the bacterial pellet in the tube.

14. Resuspend the bacterial pellet in 10 mL of TSG buffer per liter of LB culture used to generate the pellet. Add 10 mL of TSG buffer to one of the 500 mL centrifuge bottles containing the pellet and homogenize it using the glass pestle from the 15 mL glass Douncer set in combination with vortexing until the pellet is fully resuspended.

15. After the pellet has been dislodged and homogenized, transfer the 10 mL suspension using a serological pipette to the second 500 mL centrifuge bottle containing the remaining bacterial pellet. Homogenize again using the glass pestle and vortexing until the pellet is fully resuspended.

16. Once both pellets are dislodged, transfer the homogenate to the 15 mL glass Douncer by pouring it from the centrifuge bottle. If necessary, add 1–2 mL of TSG buffer to the centrifuge bottle to resuspend any remaining pellet fragments and transfer this rinse to the Douncer as well.

17. Dounce the homogenate in the 15 mL glass Douncer until all visible clumps are dispersed and the sample appears fully homogeneous.

18. Transfer the homogenate to a 50 mL Falcon tube by pouring it out of the glass Douncer. If any homogenate remains in the Douncer, add 1–2 mL of buffer to rinse it, then transfer the rinse into the same 50 mL Falcon tube.

19. Store the homogenate at -20 °C until further use.

**B. Bacterial homogenate lysis**

1. Remove the bacterial homogenate from -20 °C and thaw it on ice for approximately 30 min.

*Note: If pressed for time, the sample can be thawed in a beaker of room-temperature water for 5–10 min.*

2. Prepare the M110L microfluidizer (stored in 20% ethanol) for use:

a. Insert the glass funnel into the machine inlet.

b. Pack the bed of the microfluidizer with ice to keep the sample cold during processing.

**Figure 1.**
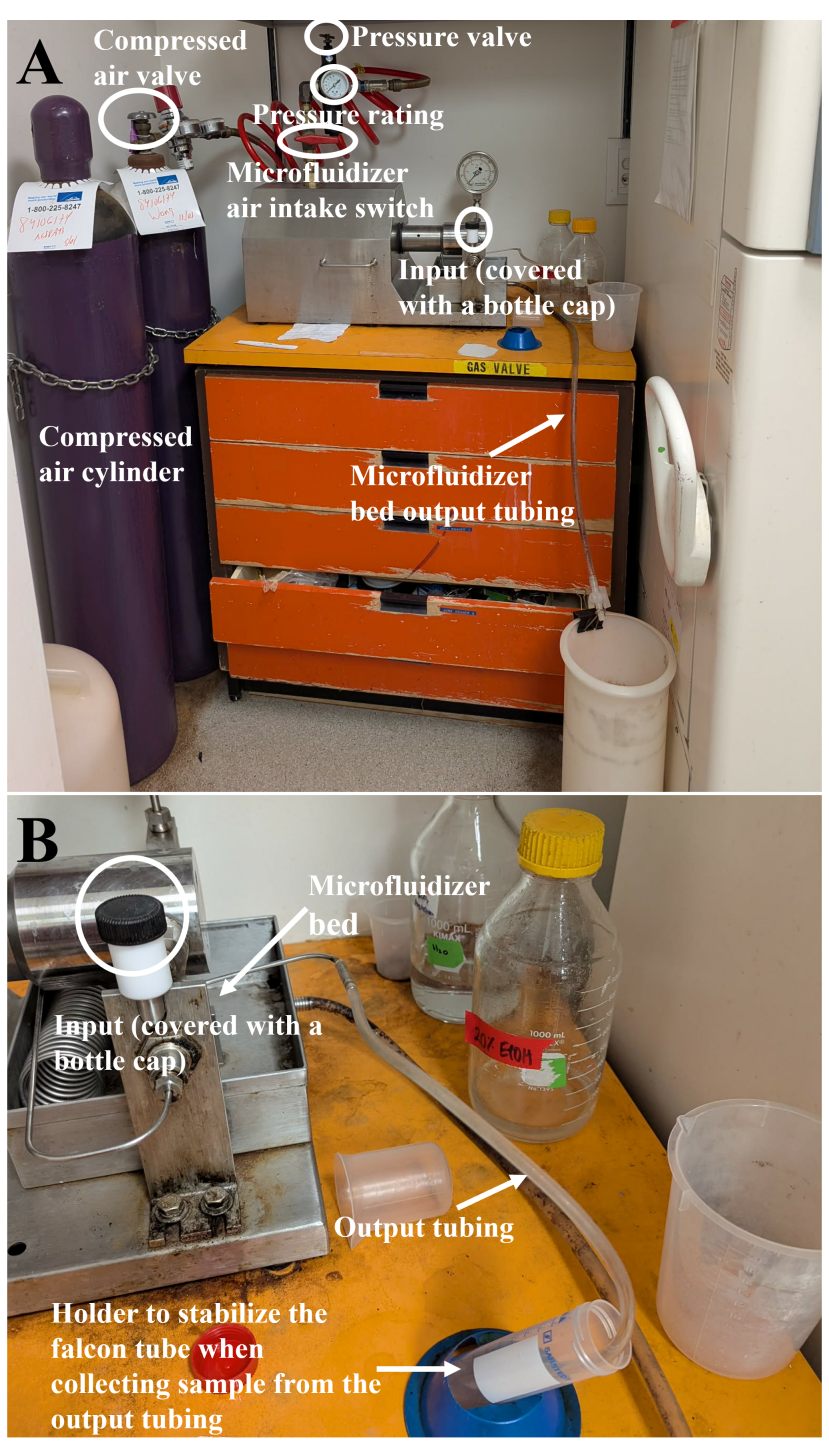
Visual overview of the microfluidizer used for bacterial cell lysis. (A) Overall setup and (B) detail of the setup.

3. Open the valve on top of the compressed air gas cylinder while keeping the microfluidizer air switch closed (Figure 1).

4. Set the microfluidizer pressure to 12,000 psi by turning the pressure valve clockwise.

5. Flush the machine with distilled water to remove the residual 20% ethanol used for storage.

a. Turn the microfluidizer air intake switch to start the machine and allow the sample to flow from the glass funnel into the microfluidizer.

b. A “click” corresponds to the sound produced when a portion of the solution in the intake valve is pushed through the machine in a stepwise manner.

c. One wash corresponds to three clicks from the machine.

d. Perform three washes with distilled water.

e. After washing, discard the excess water from the glass funnel.

**Critical:** Ensure that no air enters the machine, as air bubbles can clog the system. Always ensure that liquid is present in the glass intake funnel before turning the machine on.

6. Add the thawed bacterial homogenate to the intake funnel and pass the entire volume through the microfluidizer five times. Perform five passes through the machine in total. Pour the entire bacterial homogenate into the glass intake funnel, engage the air intake switch, and collect the output into a 50 mL Falcon tube. Once the full volume has passed through, transfer it back into the intake funnel and repeat for four additional passes.

**Critical:** Collect the output in a 50 mL Falcon tube by placing the outlet tubing into the tube. Otherwise, the sample will be directed to waste and loss.

7. After the fifth pass, add approximately 5 mL of TSG buffer to the glass intake funnel. Turn the machine on to flush any remaining homogenate from the tubing, collecting this with the rest of the lysate in a 50 mL Falcon tube. Repeat until the tubing runs clear, transferring to a new 50 mL Falcon tube as needed if the previous one reaches capacity.

8. Keep the lysed homogenate on ice.

9. Clean the machine immediately after use:

a. Perform three washes with distilled water (one wash = three clicks).

b. Follow with three washes using 20% ethanol.

c. Leave the machine filled with 20% ethanol for storage to prevent bacterial growth in the tubing.

d. Remove the glass funnel and top off the intake with 20% ethanol. Place a bottle cap over the intake to prevent the 20% ethanol from drying out.

**Critical:** If the machine runs dry, it will become unusable and require the pressure cell to be cleaned to restore proper operation.

e. Close the valve on top of the compressed air gas cylinder.

**C. Isolation of crude membrane fraction from *E. coli***

1. Transfer the homogenate into two JA-25.50 50 mL polypropylene centrifuge bottles, topping off with TSG buffer to balance the tubes. Ensure the tubes are within 1 g of each other.

2. Centrifuge using a JA-25.50 rotor in the Avanti J-E centrifuge at 4,350× *g* for 10 min to pellet the unlysed cells.

3. After centrifugation, the supernatant will contain the bacterial lysate and the pellet will contain unlysed cells. Carefully remove the supernatant while avoiding disturbance of the pellet. It is acceptable to leave a small amount of supernatant behind to ensure that no unlysed cells are collected.

4. Add an equal volume of bleach to the unlysed cell pellet and allow it to sit at room temperature for 30 min for decontamination. Afterward, dispose of the mixture down the drain and flush with running water for 15 min.

5. Transfer the bacterial lysate into TI-45 ultra-clear round-top centrifuge bottles, distributing the lysate evenly between two bottles. Top up each bottle with TSG buffer to the maximum fill volume to ensure proper balance.

**Caution:** After topping off the bottle, ensure that no more than one air bubble remains inside the tube. If multiple bubbles are present, reopen the bottle and top off again, placing the cap on carefully to minimize bubble formation. Check for bubbles by gently inverting the bottle. Limiting air bubbles helps prevent leakage during the spin, which can lead to sample loss and rotor imbalance that will stop the centrifuge.

6. Load the tubes into the TI-45 rotor. The rotor should be stored in the cold room when not in use. Place the rotor into the Optima LE-80K ultracentrifuge.

7. After placing the rotor in the centrifuge, close the lid and allow the vacuum to build. Once the vacuum reaches 750, start the run at 40,000 rpm for 45 min (corresponding to 125,440× *g*, calculated using the rotor calculator on the Beckman Coulter website).

8. After centrifugation, remove the tubes and place them on ice. Return the TI-45 rotor to the cold room for storage.

**Caution:** Inspect and clean the ultracentrifuge and rotor for any lysate that may have leaked during the spin. Failure to do so may lead to bacterial or fungal growth inside the ultracentrifuge or rotor.

9. The supernatant contains the soluble fraction of the *E. coli* lysate, while the pellet contains the membrane fraction (crude membrane).

*Note: Remove the supernatant and store it at -20 °C.*

10. Resuspend the crude membrane pellet in TSG buffer using 2 mL of TSG per liter of LB culture used to generate the crude membrane. This corresponds to 1 mL per centrifuge bottle. Add the TSG buffer, then dislodge the pellet using a P1000 pipette tip. After the pellet has been loosened, cut the end of a new P1000 pipette tip to widen the opening and pipette up and down to break up the remaining chunks until they are small enough to be easily aspirated.

11. Transfer the resuspended crude membrane to a 7 mL glass Douncer. Add 250 μL of TSG to each centrifuge bottle to collect any remaining chunks of crude membrane. Cut the end of a P1000 pipette tip and use it to transfer all remaining material to the 7 mL glass Douncer.

12. Homogenize the sample by douncing until the crude membrane suspension is uniform and no visible chunks remain.

13. Transfer the crude membrane suspension to a 15 mL Falcon tube.

14. Determine the protein concentration using the Bradford assay.

a. Add 1 mL of 1× Bradford assay solution to four semi-micro cuvettes.

b. Add 2 μL of TSG to the first cuvette (blank). Add 1 μL of crude membrane to the second cuvette, 2 μL to the third cuvette, and 3 μL to the fourth cuvette.

c. Mix each cuvette by pipetting the solution up and down with a P1000, using a fresh tip for each cuvette.

d. Place the four cuvettes into the cuvette holder of the Genesys 10 UV–Vis spectrophotometer as follows: the TSG blank in slot B, 1 μL of crude membrane in slot 1, 2 μL of crude membrane in slot 2, and 3 μL of crude membrane in slot 3.

e. On the instrument, press the *Test* button. Then press the down arrow once to highlight *Standard Curve.*

f. Name the test “Bradford.” Set the wavelength to “595 nm,” ref. wavelength correction to “Off,” and curve fit to “Linear Through Zero.” Set the number of standards to “Five,” units to “mg/mL,” sample positioner to “Auto 6,” and the number of samples to “five”.

g. Run the test.

h. Calculate the average protein concentration across the three cuvettes. Divide the value from slot 2 by 2, and the value from slot 3 by 3, to account for the 2 and 3 μL sample volumes added, respectively. Then, average the normalized values with the value from slot 1.

*Notes:*

*1. The instrument has a maximum measurable concentration of 25 mg/mL. Any reading ≥20 mg/mL should be considered outside the reliable measurement range and excluded when calculating the average protein concentration.*

*2. The Bradford assay provides an estimate of the protein concentration in the crude membrane and can be used to guide subsequent experiments, but it should not be considered an exact measurement. For assays requiring highly accurate protein quantification, protein levels should be further normalized by running and quantifying samples on SDS-PAGE gels to account for potential protein contamination.*

15. Record the protein concentration on the Falcon tube containing the crude membrane and store it at -20 °C until further use.

**Caution:** The crude membrane is stable at -20 °C for extended periods (up to ~1 year). However, protein yield decreases with each freeze–thaw cycle. It is recommended to repeat the Bradford assay after each freeze–thaw cycle to determine the updated protein concentration.

**D. Solubilization of the membrane proteome using Peptergent (PDET-1)**

1. Remove the crude membrane from -20 °C and allow it to thaw on ice for approximately 30 min.

*Notes:*

*1. If needed, the crude membrane can be thawed more quickly by placing the tube in a beaker of room-temperature water for 5–10 min.*

*2. While the crude membrane is thawing, prepare the solubilization buffer.*

2. Resuspend 100 μL of 10 mg/mL crude membrane in 200 μL of solubilization buffer and 300 μL of TS buffer (1 mg of crude membrane to 1 mg of PDET-1).

a. Adjust the crude membrane concentration to 10 mg/mL prior to solubilization so that 100 μL of crude membrane can be mixed with 200 μL of solubilization buffer and 300 μL of TS buffer, resulting in a final volume of 500 μL. If the crude membrane preparation is more diluted than 10 mg/mL, increase the volume of crude membrane added accordingly and reduce the volume of TS buffer by the same amount to compensate, maintaining a final volume of 500 μL and a total crude membrane input of 1 mg.

b. Set aside 5 μL of the 10 mg/mL crude membrane for troubleshooting by SDS-PAGE analysis.

3. Incubate the suspension on a rotisserie shaker in the cold room for 90 min to allow membrane protein extraction with PDET-1.

4. After the 90-min incubation, transfer 0.25 mL of the suspension into two separate TLA-55 1.5 mL polypropylene tubes. Visually check that the samples have equal volumes for proper balancing. If one tube has a lower volume, add TS buffer to match the volume of the other tube.

5. Place the tubes into a TLA-55 fixed-angle rotor. The rotor should be stored in the cold room when not in use.

6. Place the rotor in the Optima Max tabletop ultracentrifuge, ensuring the center of the rotor clicks securely into place before starting the spin.

7. Centrifuge at 55,000 rpm for 15 min (corresponding to 135,520× *g*, calculated using the rotor calculator on the Beckman Coulter website).

8. The supernatant contains the clarified membrane proteome extract solubilized with PDET-1, while the pellet contains the insoluble material. Set aside the pellet for SDS-PAGE troubleshooting in case your protein of interest is not successfully extracted.

9. Transfer the supernatant to a new microcentrifuge tube. Set aside 10 μL of the extract for SDS-PAGE troubleshooting.

**E. Peptide exchange from Peptergent to His-tagged Peptidisc**

1. Take 500 μL of the PDET-1 extract (1 mg PDET-1) and add 200 μL of 5 mg/mL HD-43 solution (1 mg HD-43), resulting in a 1:1 ratio of PDET-1 to HD-43 by mass. The extract volume may vary after clarification, depending on the size of the insoluble pellet or if bubbles were introduced during centrifugation due to tube imbalance. For this step, maintain the 1:1 ratio by mass of PDET-1 in the extract (2 mg/mL) to HD-43 (5 mg/mL), scaling the volumes as needed to preserve the ratio.

2. Incubate the mixture on a rotisserie shaker in the cold room for 10 min to allow peptide exchange.

**F. Affinity purification enabling exchange of PDET-1 into HD-43 and enrichment of reconstituted membrane proteins**

1. Prepare the Ni-NTA resin during the exchange step. The resin is stored as a 50:50 slurry of resin in 30% ethanol. Pipette 200 μL of the Ni-NTA slurry into a microcentrifuge tube using a cut P200 pipette tip to prevent the slurry from sticking to the tip.

a. Centrifuge at 2,000× *g* for 2 min.

b. Carefully remove the 30% ethanol supernatant, ensuring that the resin is not disturbed.

**Caution:** The resin may be aspirated when removing liquid near the pellet. If this occurs, centrifuge the tube again at 2,000× *g* for 2 min to re-pellet the resin. If additional liquid cannot be removed without disturbing the resin, leave the remaining volume in the tube and proceed to the next step.

c. Resuspend the resin in 500 μL of distilled water and incubate for 5 min at room temperature on the nutator.

d. Centrifuge again at 2,000× *g* for 2 min, then aspirate the water.

e. Resuspend the resin in 500 μL of TS buffer and keep it at room temperature on the nutator until further use.

2. Pellet the Ni-NTA resin by centrifugation at 2,000× *g* for 2 min, and then remove the TS buffer.

3. Add the 1:1 (PDET-1)/(HD-43) extract to the 100 μL of prepared Ni-NTA resin (~3 mg of sample to 100 μL of packed Ni-NTA resin) and incubate for 1 h on a rotisserie rotator in the cold room to allow binding of His-tagged Peptidisc-reconstituted MPs to the Ni-NTA resin.

**Critical:** Binding of the His-tagged Peptidisc to the Ni-NTA resin enables the selective capture of successfully exchanged and reconstituted membrane proteins in HD-43, separating them from contaminating soluble proteins and MPs whose peptide was not successfully exchanged for HD-43.

**Caution:** A total volume of 500 μL to 1 mL is recommended during incubation to allow proper mixing on the rotisserie so that the liquid moves freely with the resin. If the volume is insufficient, add TS buffer to bring the total volume to 500 μL to enable proper mixing.

**Pause point:** This step can be extended from 1 h to overnight if needed. Although the protocol specifies 1 h, this step can serve as a convenient stopping point for researchers.

4. After incubation, centrifuge at 2,000× *g* for 2 min to pellet the resin.

5. Carefully remove the flowthrough (unbound proteins) and store it at 4 °C for SDS-PAGE analysis.

6. Wash the bound Ni-NTA resin three times with TS buffer.

a. Add 1 mL of TS buffer and rotate for 5 min on the rotisserie in the cold room.

b. Centrifuge at 2,000× *g* for 2 min and then discard the wash.

7. After washing the Ni-NTA resin and removing the TS buffer, elute the bound proteins with 150 μL of TS buffer with 600 mM imidazole solution for 10 min on the rotisserie in the cold room.

**Pause point:** Similar to the PDET-1/HD-43 extract binding step to the Ni-NTA resin, this step can also be extended overnight, serving as a pause point for researchers.

8. Centrifuge at 2,000× *g* for 2 min and then carefully collect the eluate, ensuring that no resin is carried over.

9. Perform an additional centrifugation if necessary to recover any remaining eluate.

**Caution:** Multiple spins are often required to safely remove the eluate without disturbing the resin.

10. Transfer the eluate to a new microcentrifuge tube. Set aside 10 μL for SDS-PAGE troubleshooting analysis.

11. Determine the protein concentration using the Bradford assay. Use 3 μL of TS buffer containing 600 mM imidazole as the blank. For the samples, add 1, 3, and 5 μL of the eluate to 1 mL of 1× Bradford assay solution. Average the protein concentrations obtained from these three measurements. The methodology for performing the Bradford assay is described in step C14.

12. After determining the protein concentration, remove a volume corresponding to 100 μg of protein. Dilute this to a final volume of 100 μL using TS buffer, resulting in a 100 μL solution containing 100 μg of the eluted Peptidisc-reconstituted membrane proteome. Keep this solution on ice.

*Note: If the sample is too diluted to obtain 100 μg in 100 μL, use the volume corresponding to 100 μg of protein and proceed with the MS preparation steps using the adjusted volumes.*

13. Store the remaining eluted membrane proteome at -20 °C.

**Caution:** The Peptidisc-reconstituted membrane proteome can be stored for up to one week with little to no protein loss. If the sample will be used within a week, store it at 4 °C. For longer storage, keep it at -20 °C. Protein degradation can occur with freeze–thaw cycles, so the protein concentration should be re-measured by Bradford assay after each freeze–thaw cycle.

**Figure 2.**
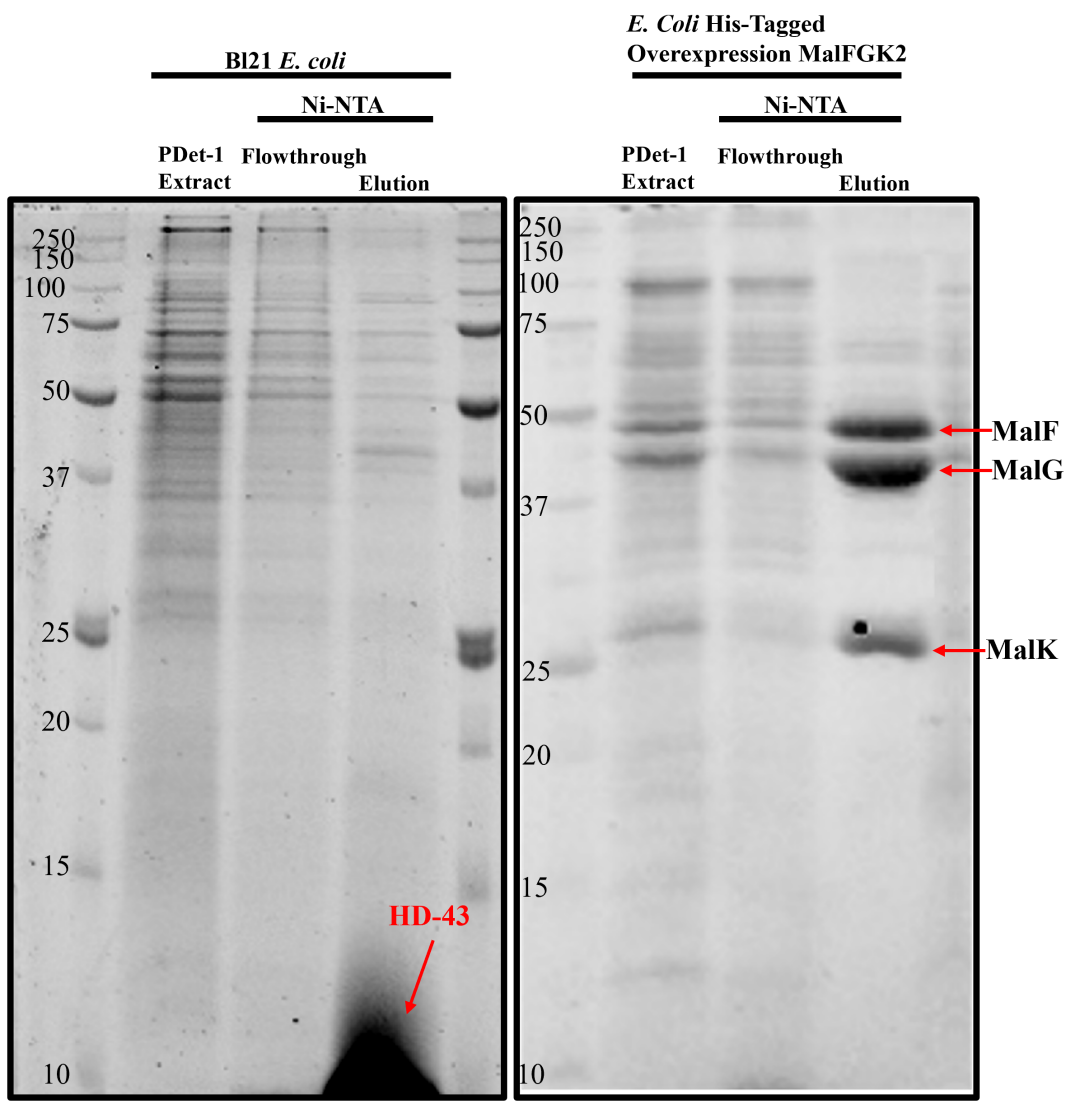
PDET-1 extraction and purification of membrane proteome and MalFGK_2_ complex. SDS-PAGE analysis of Precision Plus protein unstained protein standards, PDET-1 extract, Ni-NTA flowthrough, and elution fractions from the (left) *E. coli* BL21 membrane proteome following HD-43 reconstitution, alongside (right) the PDET-1 extract and Ni-NTA elution fractions of His-tagged MalFGK_2_.

14. Before proceeding with MS sample preparation, run the samples on SDS-PAGE to verify that the preparation was successful (example in Figure 2). If any unexpected results are observed, it is recommended to pause the protocol and troubleshoot the issue before continuing. Do not proceed to MS analysis until the problem has been resolved.

**Critical:** Based on the protocol above, run the following samples on SDS-PAGE: crude membrane, PDET-1 extract, PDET-1 insoluble pellet (only if the protein of interest is not being extracted), Ni-NTA flowthrough, and Ni-NTA elution. We recommend loading 5 μL of each sample onto the gel to prevent overloading.

**G. Bottom-up MS sample preparation**

1. Add 36.7 mg of urea to a 100 μL sample containing 100 μg of the eluted membrane proteome to reach a final concentration of 6 M urea. Vortex to mix.

2. Incubate at room temperature on the nutator for 30 min.

3. Add 1 μL of 1 M DTT to the sample to achieve a final concentration of 10 mM. Incubate on the nutator at room temperature for 1 h.

4. Add 4 μL of 500 mM IAA to the sample to achieve a final concentration of 20 mM, and incubate at room temperature in the dark on the nutator for 30 min.

5. Add 1 μL of 1 M DTT to the sample to reach a final concentration of 20 mM. Incubate at room temperature on the nutator for 30 min.

6. Add 500 μL of 50 mM ammonium bicarbonate to dilute the urea concentration to 1 M.

*Note: High urea concentrations disrupt the activity of MS-grade proteases.*

7. Add 2 μL of 0.5 μg/μL protease solution (1:100 trypsin-to-sample ratio) and incubate overnight at room temperature on the nutator.

8. The following day, add 2 μL of 0.5 μg/μL protease solution to reach a final enzyme-to-protein ratio of 1:50, and incubate overnight at room temperature on the nutator.

**Pause point:** Either of the trypsin digestion steps can be extended to incubate over the weekend, allowing researchers to pause the protocol and resume on the next working day.

**H. C18 StageTip peptide desalting and drying**

**Figure 3.**
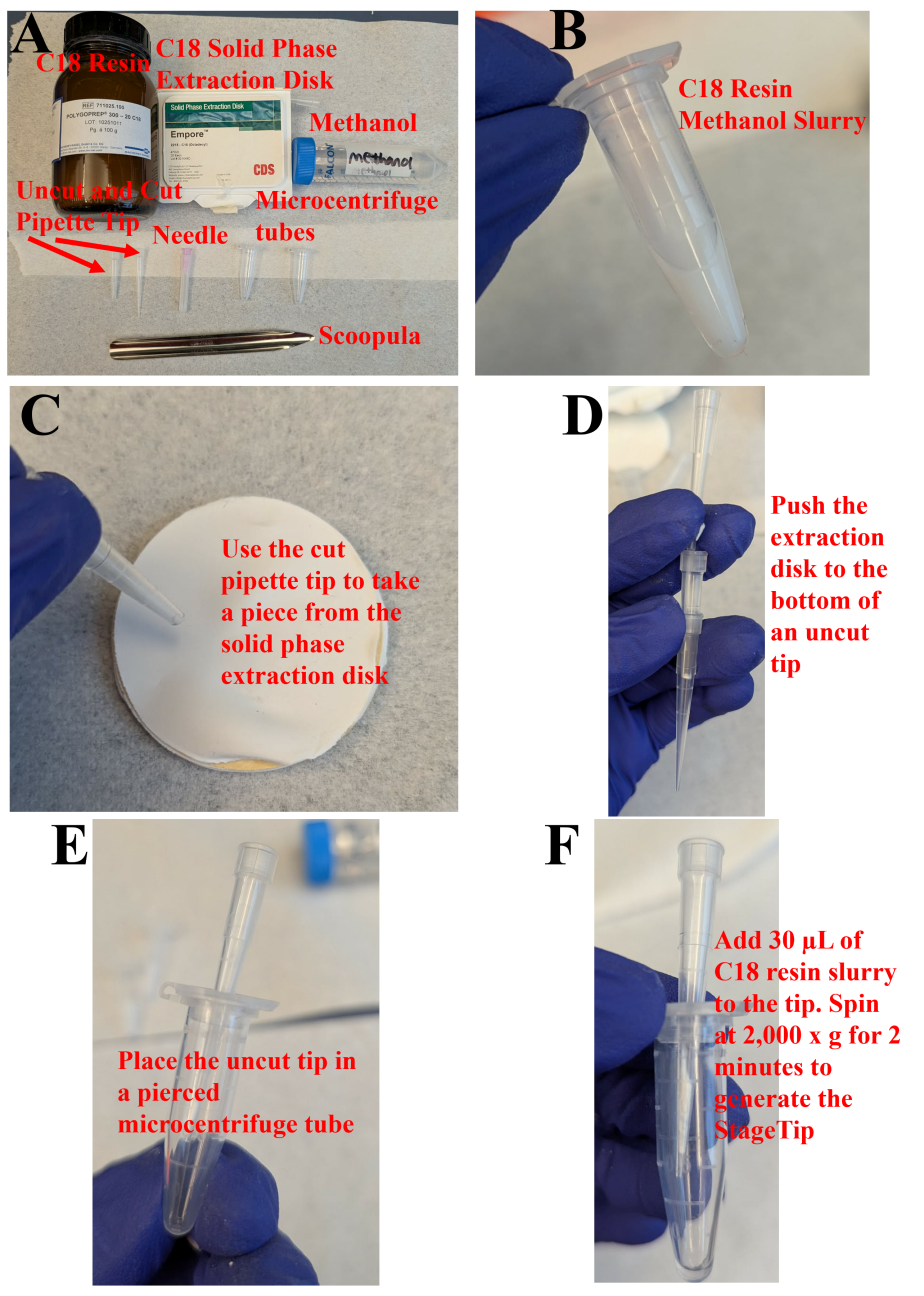
Visual workflow for preparing custom StageTips. (A) Material. (B) Resin slurry. (C) Solid-phase material. (D) Disk insertion into the tip. (E) Mounting setup. (F) Final setup.

1. Prepare the C18 resin slurry (Figure 3B), 0.1% formic acid solution, 10% formic acid solution, and 40% acetonitrile in 0.1% formic acid solution.

2. Use scissors to cut the end of a 200 μL pipette tip. Using the bottom of the cut tip, take a piece of the C18 solid phase extraction disk (Figure 3C) and transfer it into a new, uncut 200 μL pipette tip. Use a gel loading tip to push the extraction disk to the bottom of the uncut tip (Figure 3D).

3. Using a needle, punch a hole in the lid of a new microcentrifuge tube and insert the tip with the extraction disk (Figure 3E).

4. Pipette 30 μL of the C18 resin slurry into each prepared StageTip placed in its respective pierced microcentrifuge tube. Then, centrifuge at 2,000× *g* for 2 min, generating the stage tip (Figure 3F).

5. After the spin, add 75 μL of methanol and centrifuge at 2,000× *g* for 2 min.

6. Wash the StageTips twice with 100 μL of 0.1% formic acid solution, centrifuging at 2,000× *g* for 2 min after each wash.

**Critical:** If the 0.1% formic acid solution does not flow completely through the StageTip after centrifugation at 2,000× *g* for 2 min, extend the centrifugation time to 5 min. If clogging persists, discard the StageTip and prepare a new one. It is strongly recommended to verify StageTip flow before sample loading to avoid clogging issues during the sample preparation steps.

7. To the trypsin-digested samples (now 600 μL), add 20 μL of 10% formic acid solution. Check the pH of the sample and add additional formic acid if necessary to reach pH 3. Use a pH indicator strip 2.5–4.5 to check the pH.

8. Remove the StageTip from the microcentrifuge tube and pour the flowthrough from the bottom of the tube into a waste container. Then, return the StageTip to its corresponding tube.

**Critical:** Ensure the labels on the StageTip microcentrifuge tubes are clearly visible and readable, as mixing up samples at this stage could compromise over a week of work.

9. Add 100 μL of the pH 3 trypsin-digested sample to the corresponding C18 StageTip and centrifuge at 2,000× *g* for 2 min. Repeat this process, adding 100 μL of the digested sample per cycle, until the entire sample has been loaded onto the StageTip.

a. Discard the flowthrough after each spin to prevent it from contacting the StageTip. If the flowthrough level rises and begins to touch the StageTip, pour it out before continuing.

b. If the C18 resin becomes clogged, increase the centrifugal force to 3,000× *g*. If clogging persists, extend the spin time to 5 min.

c. In the worst-case scenario, use a syringe to gently force the liquid through the C18 StageTip.

10. Once the entire sample has been loaded onto the StageTip, wash the resin three times with 100 μL of 0.1% formic acid solution, centrifuging at 2,000× *g* for 2 min for each wash.

11. After the final 0.1% formic acid wash, discard the flowthrough and perform an additional dry spin at 2,000× *g* for 2 min to ensure no liquid remains in the StageTip before elution.

12. Transfer the StageTip to a new pierced microcentrifuge tube.

13. Elute the bound peptides from the StageTip with 150 μL of 40% acetonitrile in 0.1% formic acid solution.

*Note: Perform these stepwise, eluting with 50 μL of 40% acetonitrile in 0.1% formic acid solution per step and centrifuging at 2,000× g for 2 min each time, for a total of three spins.*

14. After elution, transfer the entire eluate to a new microcentrifuge tube.

15. Place the sample in a lyophilizer/dryer connected to a vacuum pump and liquid condenser and dry on low heat until all liquid has evaporated (2–4 h). Ensure the cap is open while in the machine to allow evaporation of the solvent.

**Pause point:** If needed, this step can be performed overnight. Ensure the heat is set to low if the sample is left to dry overnight.

16. After the sample has dried, check the tube to ensure no liquid remains. If the sample is completely dry, seal the tube with Parafilm around the cap and store the samples for MS analysis.

## Validation of protocol

This protocol or parts of it has been used and validated in the following research article(s):

• Antony et al. [8]. Comparative Evaluation of Solid-phase and Membrane Mimetic Strategies in Membrane Proteome Coverage and Disease-State Analysis. *Mol Cell Proteomics*. 25(2):101496. https://doi.org/10.1016/j.mcpro.2025.101496

## General notes and troubleshooting

1. This workflow is demonstrated here using *E. coli* BL21 crude membranes, but it is equally applicable to tissues and other cell types. The primary variables between sample types are the yield of crude membrane obtained from the starting material and the solubilization efficiency of the membrane proteome in PDET-1. Although not shown here, we have also successfully applied this method to mammalian cell lines and mouse organ tissues.

2. It is recommended to analyze samples by SDS-PAGE at key steps of the workflow to monitor protein recovery and sample quality. For this protocol, these steps include the crude membrane, PDET-1-solubilized crude membrane extract, insoluble crude membrane fraction, Ni-NTA flowthrough, and Ni-NTA eluent. Analyzing these samples helps determine whether the experiment proceeded correctly. One possible limitation is using insufficient crude membrane, which can result in inefficient solubilization and consequently weak band intensity on SDS-PAGE. Because PDET-1 and Peptidisc scaffolds are peptides, they will also be detected by the Bradford assay. It is therefore important to examine samples on SDS-PAGE to confirm that the preparation contains the membrane proteome, rather than the peptide scaffold alone, which could otherwise inflate the Bradford protein concentration measurement.

3. If the protein of interest fails to solubilize, resuspend the crude membrane pellet in protein loading buffer and resolve by SDS-PAGE to confirm whether the target protein remains in the insoluble fraction. If solubilization is insufficient, it may be necessary to add an additional amount of PDET-1 or employ a detergent-limited extraction as an initial step, followed by exchange into Peptidiscs. The selection of an appropriate solubilization strategy will ultimately depend on the physicochemical properties of the target protein and may require empirical optimization. We note that the optimization of extraction conditions for Peptergent-based solubilization is an active area of investigation, and it remains unclear how different conditions influence the extraction efficiency of different classes of MPs.

4. Other sample quantities can also be prepared for MS analysis. In this protocol, 100 μg of protein is processed. We recommend a minimum input of 50 μg.

5. This is a long protocol, so take care when labeling samples at each step. If performing the workflow for the first time, we recommend starting with 1–3 samples to become familiar with the procedure. Once confident with the results, the protocol can be scaled to larger sample sets for full experiments. Careful labeling is especially critical during the StageTip step, as pipetting the wrong sample into the wrong StageTip can compromise over a week of work and lead to significant reagent and sample loss.

6. Membrane proteins are stable in crude membrane preparations and in Peptidisc, so if a break is needed, it is recommended to pause the protocol and resume the following day. Leaving samples overnight in the cold room will not appreciably affect the results. It is better to proceed carefully and obtain reliable, reproducible results rather than rushing through the protocol to complete it in a single session.
